# Effect of nutrient reductions on dissolved oxygen and pH: a case study of Narragansett bay

**DOI:** 10.3389/fmars.2024.1374873

**Published:** 2024-05-23

**Authors:** Hongjie Wang, Daniel L. Codiga, Heather Stoffel, Candace Oviatt, Kristin Huizenga, Jason Grear

**Affiliations:** 1 Graduate School of Oceanography, University of Rhode Island, Narragansett, RI, United States; 2 Atlantic Coastal Environmental Science Division, United States Environmental Protection Agency, Narragansett, RI, United States

**Keywords:** nutrient management, hypoxia, ocean acidification, coastal regions, biogeochemistry

## Abstract

To assess the consequences of nutrient reduction strategies on water quality under climate change, we investigated the long-term dynamics of dissolved oxygen (DO) and pH in Narragansett Bay (NB), a warming urbanized estuary in Rhode Island, where nitrogen loads have declined due to extensive wastewater treatment plant upgrades. We use 15 years (January 2005-December 2019) of measurements from the Narragansett Bay Fixed Site Monitoring network. Nutrient-enhanced phytoplankton growth can increase DO in the upper water column while subsequent respiration can reduce water column DO and enhance bottom water acidification, and vice-versa. We observed significant decreases in surface DO levels, concurrent with a significant increase in bottom DO, associated with the nitrogen load reduction. Surface DO decline was primarily attributed to reduced intensity of primary productivity, supported by a concurrent decrease in surface chlorophyll concentrations. Meanwhile, the influence of reduced organic matter respiration led to the increase of bottom DO levels by 9 μmol kg^−1^ (approximately 0.2 mg L^−1^ for typical summer temperature and salinity) over a 15-year period, which overcame the opposite influence of oxygen reduction from solubility decreases due to warming temperatures. In contrast, long-term changes in surface pH have not exhibited discernible trends beyond natural variability, likely due to the complex and sometimes opposing influences of biological activity and changing river flow conditions. We observed a slight increase in bottom pH, associated with the increase in DO in bottom water. Notably, future variations in freshwater discharge, particularly linked to extreme precipitation events, may further influence water carbonate chemistry and thereby impact pH dynamics. This study highlights the necessity of long-term time series measurements in helping understand the impacts of environmental management practices in improving water quality in coastal regions during a changing climate.

## 1 Introduction

Coastal marine ecosystems are among the most ecologically and economically productive areas on the planet, providing ecosystem services that range from commercial and recreational fisheries to cultural enrichment and support of human well-being (Costanza et al., 1997). Dissolved oxygen (DO) is a critical indicator of system health, because low concentrations have profound impacts on marine living resources and key biogeochemical processes. The threshold at which estuarine fauna are negatively impacted varies greatly across species, but major detrimental impacts often occur at DO below 3 mg L^−1^ (hypoxia; Vaquer-Sunyer and Duarte, 2008). At a basic level, hypoxia is driven by organic matter respiration which, in strongly seasonal and temperature-stratified systems, tends to be decoupled in space and time from production (Cloern and Jassby, 2008; Rabalais et al., 2010). Nutrient-fueled eutrophication has generally been the major driver of coastal hypoxia, and efforts are ongoing in several coastal systems to mitigate eutrophication through reduction of external nutrient loading, particularly from wastewater treatment facilities (WWTF) (Diaz and Rosenberg, 2008; Boesch et al., 2009; Boesch and Goldman, 2009; Taylor et al., 2011, 2020; Greening et al., 2014; Staehr et al., 2017; Lefcheck et al., 2018; Boesch, 2019; Rabalais and Turner, 2019; Codiga et al., 2022). Nutrient load reductions reduce hypoxia by decreasing primary production and the subsequent delivery of organic material to bottom waters. In the US, successful load reductions have occurred in Tampa Bay, Boston Harbor, Chesapeake Bay and Narragansett Bay (NB), with generally positive results for seagrass recovery and hypoxia decline (Lefcheck et al., 2018; Taylor et al., 2020).

Atmospheric carbon dioxide (CO_2_) has increased from the preindustrial period of 280 ppm to ~420 ppm in 2023, and is expected to continue increasing unless global action is taken to curb emissions. Throughout the history of human emissions, the oceans have performed an essential service by absorbing 25% of all CO_2_ emissions since the Industrial Revolution (Friedlingstein et al., 2022). The continuous uptake of CO_2_ by seawater causes a process known as ocean acidification (OA), whereby a cascade of chemical reactions causes an increase in acidity and alters the forms and availability of inorganic carbon to marine life. OA is characterized by increased partial pressure of carbon dioxide (*p*CO_2_) and decreased pH and calcium carbonate saturation state (Orr et al., 2005). OA has a significant and lasting impact on marine organisms, as acidified water can increase the thermodynamic cost of secretion and maintenance of calcareous structures in ecologically important organisms, such as pteropods and coccolithophores (Kleypas et al., 2019; Bednaršek et al., 2021). After multiple decades of high precision monitoring of OA throughout the global oceans, the anthropogenic causes of this open ocean phenomenon have been well described.

Local processes occur concurrently with atmospheric-driven OA (Cai et al., 2021). Due to the coexistence of various regional processes, the range of pH change rates in coastal systems can be orders of magnitude wider than in the open ocean (Waldbusser and Salisbury, 2014). The mixing of coastal waters with freshwater plays an important role in the distribution of alkalinity and dissolved inorganic carbon (DIC). Freshwater is naturally low in alkalinity and DIC, and as a result, the mixing of freshwater with coastal seawater reduces buffer capacity in low salinity regions (Rheuban et al., 2019; Pimenta et al., 2023). This effect is especially pronounced after episodic storm events, but it is also shaped by land-based chemical weathering and local biogeochemical processes (Osborne et al., 2022). We can predict how water mass mixing affects ocean acidification based on a two-endmember water mass mixing model. When the measurements differ from what we expect from this water mass mixing we can conclude that other factors, such as biological processes and/or fluxes across the air-sea interface, play a significant role (Cai et al., 2021).

Studies have confirmed that the combination of physical and biological processes in coastal ecosystems causes strong diurnal variability in carbonate chemistry: the diurnal amplitudes for *p*CO_2_ and pH in many shallow coastal areas are 10~100 times higher than that in the open ocean (Carstensen and Duarte, 2019; Rosenau et al., 2021; Torres et al., 2021). This high temporal variability creates significant challenges for detecting OA changes (Carter et al., 2019). Among all driving processes, eutrophication and extreme weather events strongly exacerbate water OA (Wallace et al., 2014; Carstensen and Duarte, 2019; Wang et al., 2020; Cai et al., 2021; Guo et al., 2022). However, key knowledge gaps remain in our understanding of the impact of OA in coastal ecosystems where seawater carbonate chemistry is far more variable (Hofmann et al., 2011). Based on scaling of biological responses to the population level, annual repetition of seasonal pH excursions have the potential to cause large declines in the abundance of bivalves in estuaries of the northwest Atlantic region (Grear et al., 2020).

Thanks to the aggressive management actions that have been implemented throughout the NB watershed, including nitrogen reduction upgrades at eleven WWTFs within Rhode Island, the total nutrient reduction from prior 2005 to 2012 was greater than 50% (Oviatt et al., 2017) and the mean bay-wide nitrogen loading to NB in 2013–2019 was 34% less than the mean loading from 2005–2012 (Codiga et al., 2022). The primary productivity and occurrence of hypoxia has markedly declined after the application of these managed nutrient reductions (Codiga et al., 2022). Nutrient reduction should theoretically alleviate OA in bottom water because of reduced organic carbon respiration. However, there has been a decrease in the magnitude of winter-spring blooms that has multiple potential causes, including increased overwintering of grazers (Oviatt et al., 2002, 2017); Oviatt et al. (2022) nonetheless considered reductions in nutrient loading to be the primary driver of this change. Regardless of the cause, this change in bloom dynamics may dampen biological OA regulation in the winter season.

The aggressive nutrient reduction provides a rare opportunity to understand the feedback of OA to intentional decadal nutrient reduction in the context of global warming and climate change (Codiga et al., 2022). Codiga (2020) has reported that in NB, near-bottom water (0.1°C yr^−1^) was warming twice as fast as the surface (0.05°C yr^−1^) and suggested the unexpected faster warming trend near the bottom has less to do with local air sea interaction and the influence of warming air temperatures and is more likely the result of the intrusion of deep shelf waters (Pfeiffer-Herbert et al., 2015) that have warmed as a result of the warming in northwest Atlantic shelf water (Wu et al., 2012; Gonçalves Neto et al., 2021). The rapid warming and intrusion of deep water will impact the solubility of oxygen in seawater, which is a function of temperature and salinity. Salinity change has a secondary influence on DO solubility compared to warming in NB (Codiga, 2020). However, the impact of offshore warming, and hydrological processes changing river flow, on OA in NB has not yet been quantified and it is urgently needed to fill in these knowledge gaps to forecast ecosystem changes under climate change.

Our analysis centers on inter-annual to decadal time scales, the long-term trends across all years of observations. From about April through September, Narragansett Bay is density-stratified and consists mainly of two layers with distinct water quality characteristics (e.g., Codiga, 2012). We therefore frame our hypotheses in terms of surface and near-bottom conditions, with one focused on oxygen and the other on OA, as follows: H1a. Surface DO is dominantly influenced by the primary productivity declines due to nutrient load reduction; H1b. Near-bottom DO will increase because of reduced organic matter respiration, despite decreasing solubility resulting from warming; H2a.Surface OA is dominated by atmospheric CO_2_ dissolution and warming, but can be influenced by primary productivity; H2b. Near-bottom OA will be slowed down (pH increase) by reduced organic matter respiration even though warming and anthropogenic CO_2_ dissolution has the potential to decrease pH.

To examine the hypotheses, we revisited observations from the ongoing Narragansett Bay Fixed Site Monitoring Network (NBFSMN) starting from 2001, quantified the decadal DO and OA changes, and applied a two end-members mixing model analysis to examine the impacts of physical, chemical, and biological processes on carbonate chemistry. Because NB is one among many temperate urbanized estuaries, the scientific outcomes will be applicable for other regions subject to multiple environmental stressors and intentional nutrient loading reduction.

## 2 Methods

### 2.1 Observations

**FIGURE 1:**
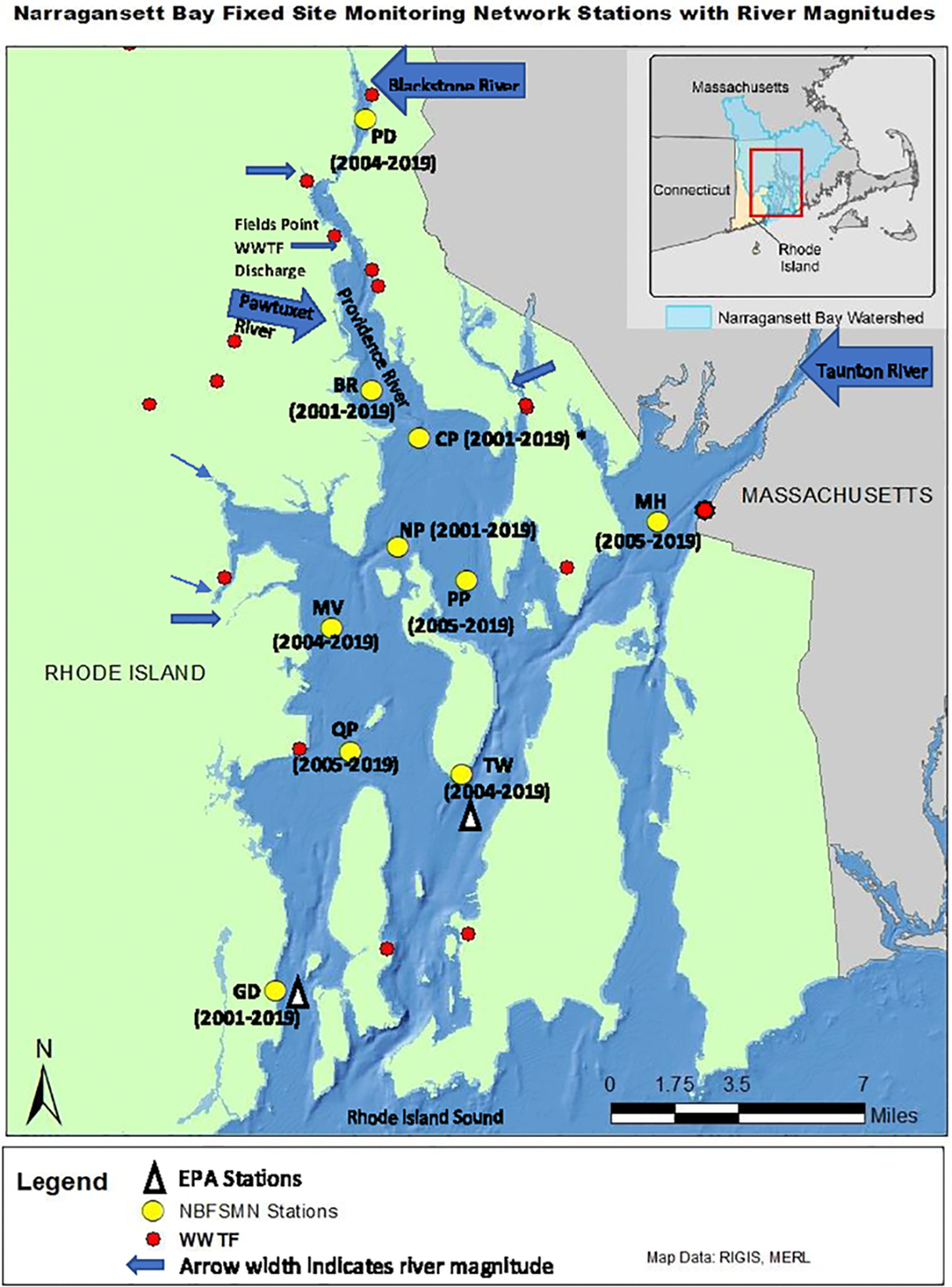
Narragansett Bay Fixed Site Monitoring Network sites (yellow dots), annotated with station abbreviations and years of data available (CP, Conimicut Point, has all years except 2004) for analysis in this study. Arrows show river nutrient source locations with sizes scaled by long-term mean river flow volume. Red dots are point source (WWTF) nutrient input locations. The white triangles show the two EPA monthly monitoring sites in Pimenta et al. (2023).

To assess NB water quality a number of agencies have worked together to establish a fixed-site monitoring network (NBFSMN, yellow circles in Figure 1), which is now an essential component of Rhode Island’s bay monitoring strategy. The stations were located strategically to focus on low water quality regions of upper NB and serve as sentinels of changing conditions. There is a greater concentration of sites in upper NB to help address influences of discharges from both wastewater treatment facilities and large tributary rivers there. The Rhode Island Department of Environmental Management (RI-DEM) Office of Water Resources has taken a lead role in coordinating the multi-agency network effort. Monitoring stations in the network are fitted with YSI (Yellow Springs International; Yellow Springs, Ohio, USA) multi-parameter instrumentation that collects water quality data on a continuous basis (usually every fifteen minutes). The dataset includes temperature, salinity, total Chlorophyll (Chl), DO, depth, and pH. The pH values are measured on the National Bureau of Standards (NBS) scale, which is used for all results presented. We converted DO from mg L^−1^ to μmol kg^−1^ using *in situ* temperature and salinity to discuss the absolute change of DO in any given water mass. All datasets used have been subjected to the network’s QA/QC review (Stoffel and Langan, 2019; RIDEM, 2020). All datasets are hosted through the RIDEM website (https://dem.ri.gov/environmental-protection-bureau/water-resources/research-monitoring/narraganset-bay-assessment-0).

The initial focus of the monitoring network was hypoxia, not ocean acidification, and the precision of pH measurements is relatively low (± 0.1). Nonetheless, analysis of this long-time monitoring data provided an opportunity to estimate the overall carbonate chemistry changes in NB from 2001 to the present, helping narrow the OA knowledge gap in the U.S. Northeast (Rheuban et al., 2019).

### 2.2 Data preparation

**FIGURE 2:**
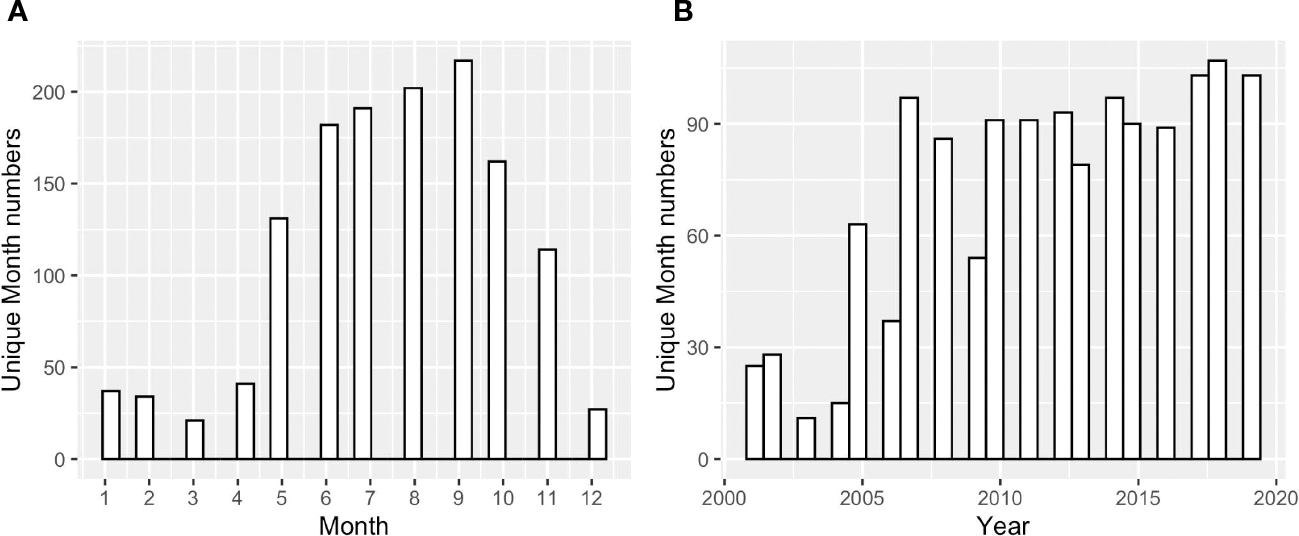
**(A)** The counts of sampled months across Narragansett Bay at surface and bottom, over all years 2005 to 2019; **(B)** the counts of sampled months across different sampling years across the entire bay at surface and bottom.

To examine the annual-mean bay-wide average DO and pH changes on interannual to decadal timescales, we analyzed stations with at least 13 years of observations. There were 10 stations that match this criterion (Figure 1). Most stations were sampled from May to October, with two (GD, TW, Figure 2) that were sampled year-round. Most time series started from 2005 (formal start of NBFSMN), when major managed nutrient reductions began to be implemented, while four stations (GD, BR, NP, CP) have data dating earlier than 2005. Therefore the analysis was more robust for changes occurring in the summer, and for years after 2005.

**FIGURE 3:**
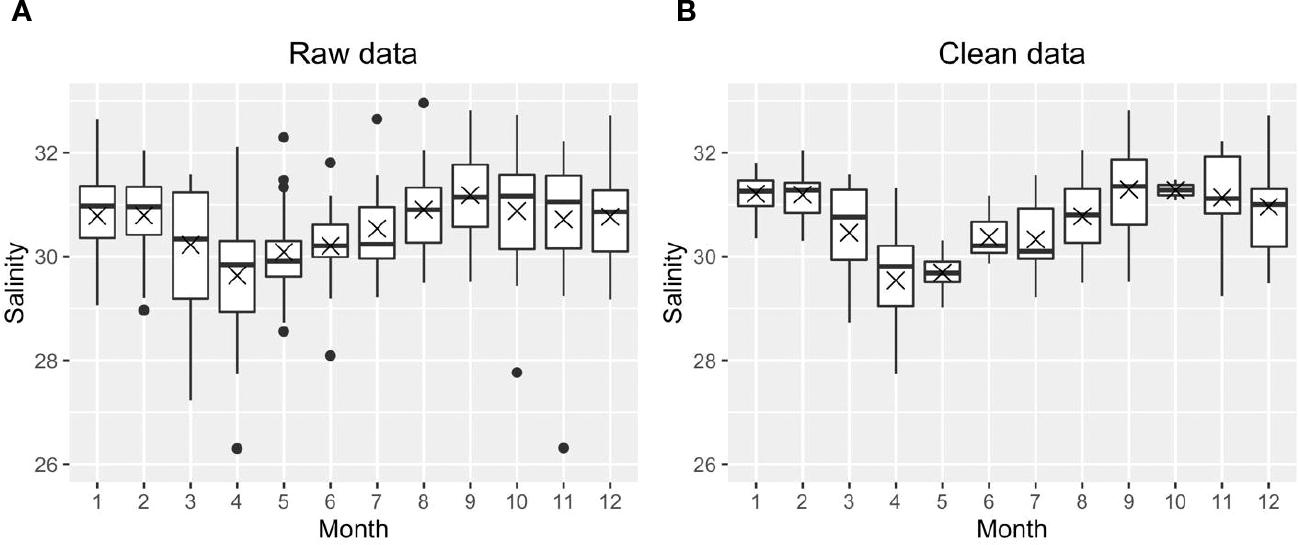
An example to show the distribution of raw monthly salinity data **(A)** and clean monthly salinity data without outliers **(B)** at Station GD. The cross and horizontal lines are the medians and means, respectively. Note, the boxes cover the 25^th^ to 75^th^ percentiles in each month. The mean is indicated by the “X”, and the median (2^nd^ quartile, or 50^th^ percentile) is marked as the solid horizontal line within the box. The vertical lines (whiskers) extend to the smallest and largest values that are not considered outliers, the 1.5 times the IQR from the quartiles.

We first calculated the daily means using the raw dataset, then all daily mean outliers were removed for each parameter (temperature, salinity, DO, pH) based on site criteria at each depth. Outliers were removed because short but extreme events, such as episodic phytoplankton blooms or extreme hypoxia, may skew the mean status if included in the mean state analysis. Outliers were defined as either higher than Q3 + 1.5* IQR or smaller than Q1−1.5*IQR (IQR: interquartile range; site, depth, and parameter dependent). Figure 3 shows one example before and after removing the outliers for salinity at surface Station GD. All the black dots in Figure 3A were defined as outliers during this round of testing; the entire row of data (all parameters) was removed if any individual parameter, i.e., temperature, salinity, DO, pH was categorized as an outlier. We repeated the same process for each of the four parameters (temp, salinity, DO, pH) until there were no outliers for any parameter (Figure 3B). We did not remove any potential outliers for Chl because there were no Chl records in bottom layers; including Chl in this treatment would introduce artificial biases between the surface and bottom layer. This data treatment removed 28% of raw data across nine stations (note that PD was not included because of its much lower salinity compared to other stations). The monthly means were then calculated using the ‘clean’ daily mean, or the daily mean after removing the outliers.

### 2.3 Inorganic carbon system

The carbonate system is commonly described by four principal components: total alkalinity (TA), dissolved inorganic carbon (DIC), pH, and carbon dioxide partial pressure (*p*CO_2_). Measurement of any two parameters in addition to temperature, pressure, and salinity (as well as minor bases such as phosphate and silicate) can fully constrain all other equilibrated carbonate system components (Millero, 2007; Dickson, 2010; Byrne, 2014). We used the R ‘seacarb’ package (Gattuso et al., 2021) for these calculations. TA represents the ability to neutralize acids to the equivalence point of carbonate or bicarbonate (Dickson, 1981), thus, TA is nearly orthogonal to pH, and mixes linearly, as it is unaffected by temperature, gas exchange, or anthropogenic CO_2_ absorption. Therefore, TA and pH pairs are an ideal carbonate system parameter combination to estimate other parameters (Emerson and Hamme, 2022), such as DIC in this study.

Based on three years’ monthly observation, Pimenta et al. (2023) developed an empirical relation to calculate TA from salinity in Narragansett Bay (TA= 477.62 + 51.99 * Salinity, adj. R squared is 0.82). We used this relationship to determine the TA from measured salinity, and then calculated DIC using the measured pH and salinity-estimated TA for any given site and depth. With the standard error of calculated TA (± 10 μmol kg^−1^) and measured pH (± 0.1), the error propagation of calculated DIC averaged 34 μmol kg^−1^. This error constituted less than 2% of the absolute DIC values in areas with salinity between 20 and 32. Our main focus is not on absolute values of DIC, but rather on its variability in response to mixing processes (as explained in Section 4.2.1), which showed changes > 600 μmol kg^−1^ within this salinity range—an order of magnitude larger than the propagated error.

**FIGURE 4:**
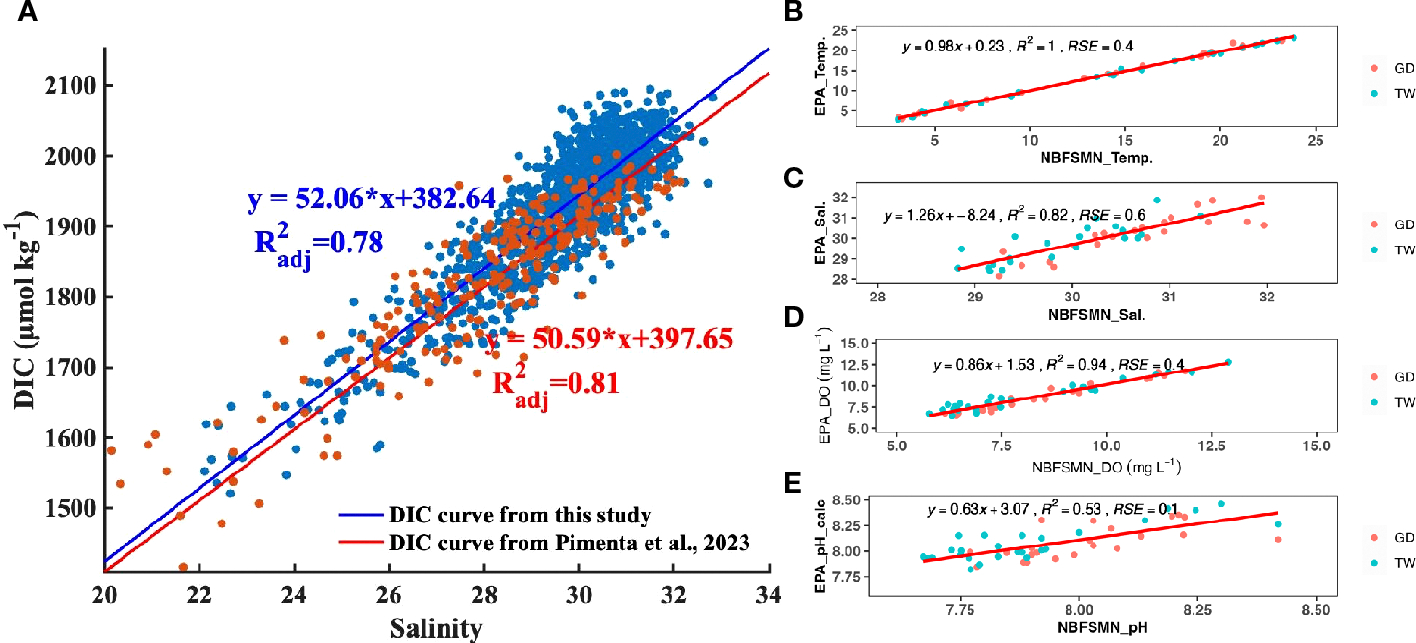
**(A)** The distribution of DIC as a function of salinity. Each DIC point was calculated with TA (TA= 51.99 * salinity+477.62) and the measured pH (NBS scale). The blue straight line and linear regression is the fitted curve from this study. The red line is the reported DIC vs salinity regression from Pimenta et al. (2023). **(B–E)** are regressions between the EPA and NBFSMN datasets for temperature, salinity, DO, and pH respectively, showing the residual standard error (RSE).

The relation between salinity and calculated DIC (using TA from the Pimenta et al. (2023) salinity-TA relationship and the measured pH) is DIC=52.05*salinity+382.64, (R^2^_adj_=0.79), close to the empirical salinity-DIC relationship that Pimenta et al. (2023) derived from discrete bottle samples (DIC=50.60*salinity+397.5, R^2^_adj_ =0.81). The consistency between these two DIC datasets (Figure 4) confirms the data quality of continuous pH observations used here. The DIC difference calculated by these two DIC vs. salinity regression lines ranged from 17 μmol kg^−1^ to 32 μmol kg^−1^ when salinity ranged from 22 to 32. This range of DIC variation falls within the expected error propagation (noted above) attributable to the uncertainties in pH measurement and TA prediction. Consequently, despite having just a single directly measured carbonate system parameter—pH—we can investigate the carbonate chemistry system using that together with the calculated DIC.

Additionally, we compared pH at the two NBFSMN sites with annual measurements, GD and TW, against independent EPA monthly monitoring nearby (stations shown in Figure 1) from 2017 to 2019. We found the residual standard error (RSE) between these two sources: temperature differed by 0.4°C, salinity by 0.6 PSU, and DO by 0.4 mg L^−1^. Using the calculated pH from surface total alkalinity and DIC from lab-analyzed discrete samples (Pimenta et al., 2023), the RSE for pH was 0.01. Even though variations in the calculated pH were more marked (Figure 4E), they stayed within the anticipated uncertainty range of ±0.1. This evidence reinforces the integrity of the continuous measurements used here.

### 2.4 Statistical method

After removal of outliers (section 2.2), we averaged the daily means for each station (s), layer (l), month (m) and year (yr). Taking pH for example, the climatological seasonal cycle $\left(y_{\text{seas}}\right)$ was derived by averaging each of the 12 months over the multiyear time series at different layers and stations $\left({\bar{pH}}_{s,l,m}\right)$ : The seasonal cycles were removed from the monthly means using the approaches described in detail in Takahashi et al. (2009) and Stoffel and Langan (2019). Thus, the de-seasonalized (denoted with superscript “ds”) values used in remaining analyses were computed as $pH_{s,l,m,yr}^{ds}=pH_{s,l,m,yr}-{\bar{pH}}_{s,l,m}$. This method produced a time series of de-seasonalized monthly anomalies $\left(pH_{s,l,m,yr}^{ds}\right)$, or the monthly residuals after removing the climatological seasonal cycle. The trend of monthly anomalies represented the long term change at each station, each layer. We used Durbin-Watson Test to examine whether there was autocorrelation in the monthly anomalies, and found that p value in 89% of time series was > 0.05, suggesting that there was no significant correlation in 68 out of 78 trends analysis. Therefore, to make the method consistent, we did not add autocorrelation in the final trend analysis. We then averaged the monthly anomalies across all nine stations to get the bay-wide monthly anomalies ${\bar{pH}}_{l,m,yr}^{ds}$ (here the overbar denotes bay-wide) and its trend. We used the same method to compute bay-wide monthly anomalies for temperature, salinity, DO and Chl and their trends.

## 3 Results

### 3.1 Bay-wide DO and pH distributions

**FIGURE 5:**
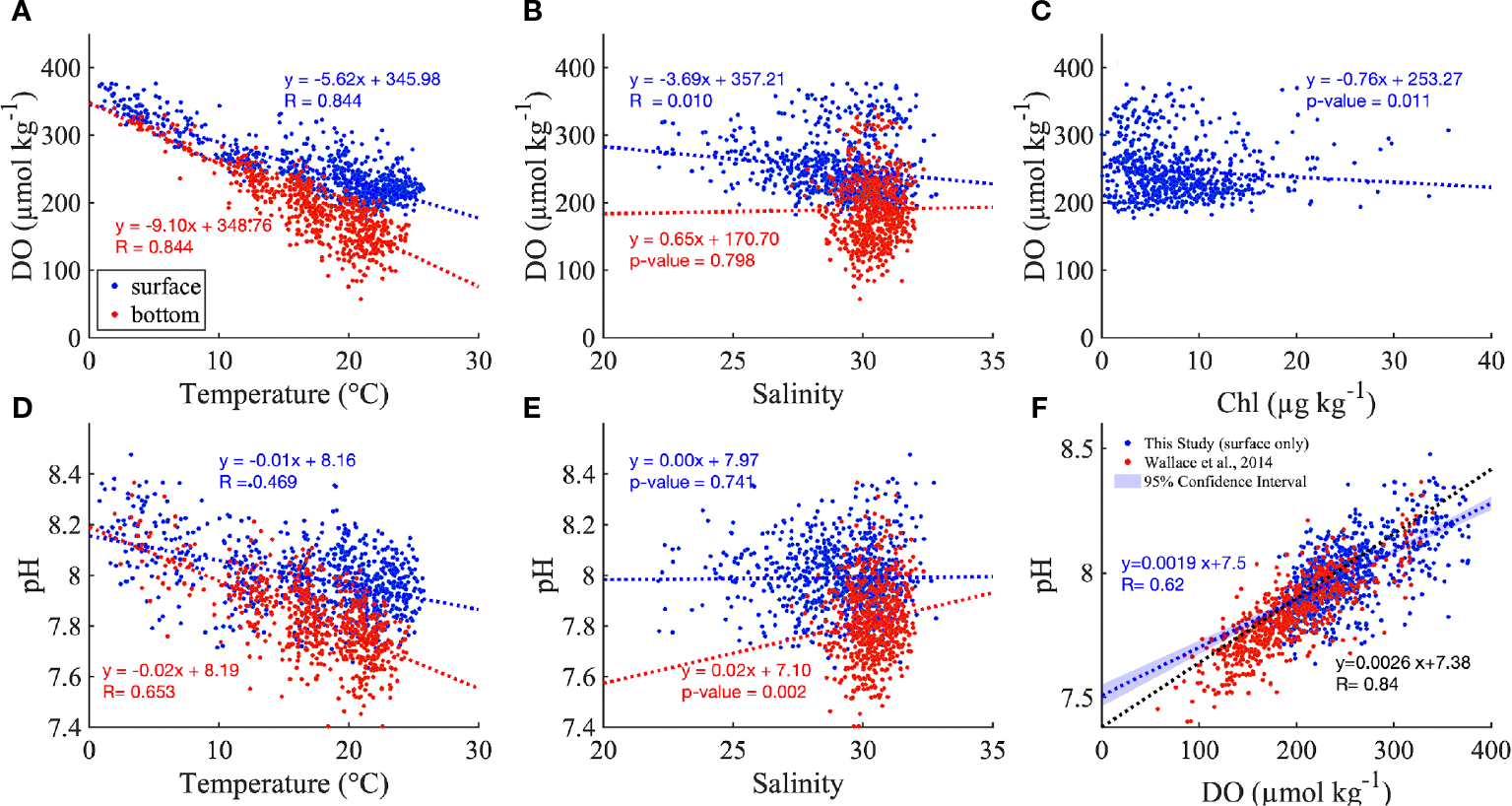
The distribution of DO along temperature **(A)**, salinity **(B)** and Chl **(C)**. The distribution of pH against temperature **(D)** and salinity **(E)**, and DO **(F)** for all monthly mean values across the nine stations. The red and blue dashed lines are the best fitted linear regression for surface and bottom in **(A–E)** The inserted regressions show the best fitted linear regression with p value or R value (when p value<0.001). The blue and red dots represent the surface and bottom measurements, but the insert equations in **(F)** are the best fitted linear regress between DO and pH for surface only (in blue), for comparison to the regression reported by Wallace et al. (2014) in Narragansett Bay (black dashed line) which is based on surface summer measurements. Note, to put the Wallace et al. (2014) relationship on the same scales as our analysis, we added 0.13 to its y-intercept (Badocco et al., 2021) to convert from the total pH scale to the NBS pH scale, and converted the DO from mg L^−1^ to μmol kg^−1^.

Both DO and pH were negatively correlated with temperature, and the slope of the relationship was more negative in the bottom layer than in the surface water (Figures 5A, D). DO significantly decreased with salinity in the surface layer (Figure 5B). There was no clear relationship between DO and Chl in the surface layer (Figure 5C), in part due to the scattered distribution of DO in low salinity ranges, which occur at landward locations most influenced by rivers. pH increased with salinity in the bottom layer, while this relationship was not significant in the surface layer (Figure 5E).

The consistency of the pH vs DO relationship in both surface and bottom layers (blue and red, respectively, in Figure 5F) confirmed the dominant role of biological processes rather than air-sea exchange, which if it was more important would influence surface results more strongly and lead to difference between surface and bottom results. The pH vs DO relationship from this study based on bay-wide sensor time series was close to the one reported by Wallace et al. (2014) based on surface data from vertical summer profiles in NB (Figure 5F, blue and black dashed lines, respectively), especially when DO ranged from 150 to 250 μmol kg^−1^. The differences mainly result from the different nature of the datasets; for example, the longer duration and bay-wide spatial coverage in this study spanned a larger range of salinity, temperature and DO compared to the summer-only sampling in Wallace et al. (2014).

### 3.2 Observed long-term trends

#### 3.2.1 Temperature and salinity

**FIGURE 6:**
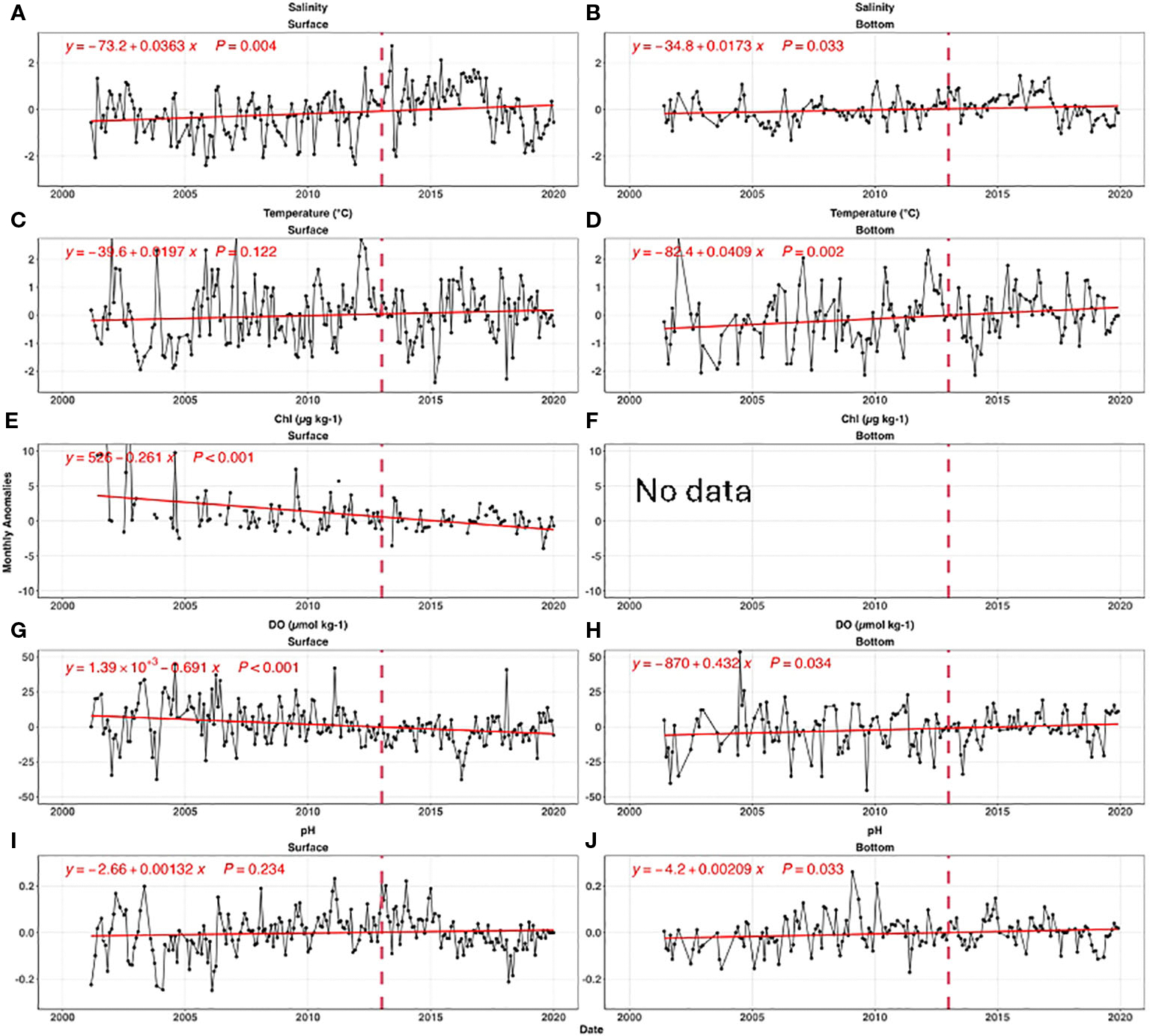
The time series of bay-wide monthly anomalies for salinity **(A, B)**, temperature **(C, D)**, chlorophyll **(E, F)**, DO **(G, H)**, and pH **(I, J)**. The left and right panels are for surface, and bottom, respectively. The red lines are the best fitted linear curves, and insert equations are the best fitted linear regression. The vertical dashed line in each panel shows 2013, when 50% of nitrogen load reductions was completed. Panel **(F)** was intentionally left blank because there was no bottom chlorophyll measurement. Note, for simplification, we only present the bay-wide monthly anomalies; the same analysis based on individual sites with similar results can be found in Supplementary Figure S1.

Figure 5 shows bay-wide monthly anomalies for all parameters relative to the seasonal cycle. River flow, a dominant influence on salinity, has wide inter-annual variations due to hydrological conditions (e.g., Codiga et al., 2022). Bay-wide average salinity was stable (p value=0.31) before 2011, then increased ~2 units from 2011 to 2016 (p value=0.003) (Figure 6A). Surface salinity quickly decreased by 2 from 2016 to 2019. The bottom salinity was less variable than the surface, but followed a similar temporal pattern. The high salinity from 2014 to 2016 was tied to the low river flow during this period (Codiga et al., 2022).

The temperature at surface and bottom increased significantly with a rate of 0.02°C yr^−1^ (p value= 0.12) and 0.04°C yr^−1^(p value=0.002), respectively (Figures 6B, C). The warming rates were higher than global means, perhaps due to changes in offshore water circulation impacting the water entering the deep East Passage (Codiga, 2020). Our absolute rates differ modestly from the results of Codiga (2021), most likely due to differences in analysis methods, but the faster warming rate in bottom water is consistent.

#### 3.2.2 Surface chlorophyll

Surface chlorophyll exhibited a significant decline with a trend rate of 0.26 μg L^−1^ yr^−1^ (p value=<0.001, Figure 6E), largely because of nutrient load reductions due to WWTF upgrades completed by 2014. Because there are no bottom chlorophyll measurements, we consider surface chlorophyll a proxy for sinking labile organic carbon.

#### 3.2.3 DO

Surface DO decreased from 2005 to 2015 (Figure 6G), and then slightly increased after 2016. Over the full timeseries the decrease was significant at a rate of 0.69 μmol kg^−1^ yr^−1^(p value<0.001). The bottom DO consistently increased at a rate of 0.43 μmol kg^−1^ yr^−1^ (p value=0.034, Figure 6H).

#### 3.2.4 pH

The surface pH had the opposite trend as DO; it increased from 2005 to 2013, and then decreased after 2013. Overall, there was no significant pH change in the surface (p value =0.234), while bottom pH significantly increased (+0.0021 yr^−1^, p value =0.033) across the time series in spite of decreasing pH after 2017 (Figure 6J).

## 4 Discussion

The DO consumption in the bottom layer generally comes from biological respiration of autochthonous organic carbon as reported in other coastal systems (Caballero-Alfonso et al., 2015; Fennel and Testa, 2019; Pitcher et al., 2021). Codiga et al. (2022) suggested that in NB the response of the ecosystem to managed nutrient reduction has been “textbook”, in the sense that chlorophyll and hypoxia declined from about 2005 onward, especially after 2013 when the nitrogen load declined markedly relative to earlier years with comparable river flow. We also interpret the DO patterns to be the result of decreases in the supply of fixed carbon, associated with reduced phytoplankton that fuels deep DO consumption and hypoxia. While Codiga et al. (2022) examined the temporal changes of extreme conditions (hypoxic events and high-chlorophyll events), here our data treatment—removal of 28% outliers—has excluded most of the extreme events. Instead, we quantify the temporal change of de-seasoned monthly-mean chlorophyll, DO and pH. Therefore, when comparing the two studies, we should keep in mind the difference in temporal periods (annual as available, vs summer) and data treatment (monthly mean, vs extreme values). Even though the datasets and treatments were different they gave similar results: Codiga et al. (2022) found significantly decreased high-chlorophyll events and decreased hypoxia duration and strength, and our results show decreased mean chlorophyll and increased mean bottom DO. We also did the same analysis for summer months only (June to September, not included here), and found the temporal trends were almost the same, which is reasonable given the eutrophication and hydrological changes are mostly limited to the summer months.

### 4.1 Processes impacting long term DO changes

#### 4.1.1 Surface DO is mainly controlled by the biological effect of declining chlorophyll

**FIGURE 7:**
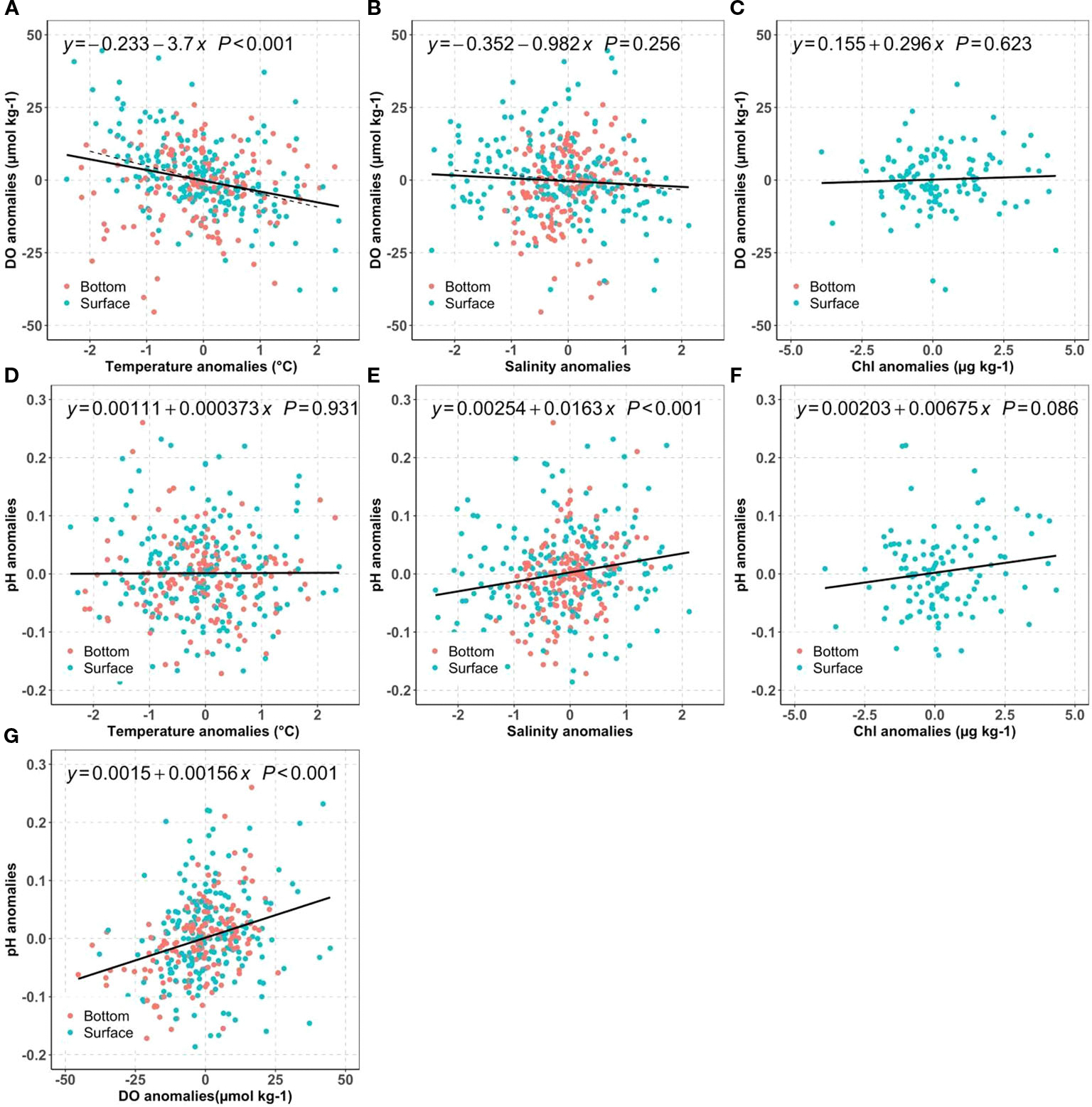
Distribution of bay-wide monthly mean DO anomalies against temperature **(A)**, salinity **(B)**, chlorophyll **(C)**; distribution of bay-wide monthly mean pH anomalies against temperature **(D)**, salinity **(E)**, Chl **(F)**, and pH anomalies vs DO anomalies **(G)** for the surface (blue) and bottom (red). The black lines show the best linear regression fit for averaged surface and bottom values. The dashed line in **(A, B)** are the theoretical regression line for DO saturation against temperature or salinity for a typical Narragansett Bay water (T=17°C and S=30).

We first investigated how much of the mean DO changes can be attributed to long-term warming, salinity change, and nutrient reductions by examining the DO anomalies against temperature (Figure 7A), salinity (Figure 7B) and Chl (Figure 7C). For typical NB seawater (S=30, T=17°C), the DO solubility decreases by 4.8 μmol kg^−1^ for 1°C warming, and 1.6 μmol kg^−1^ for 1 PSU salinity increase. Because we do not expect major differences in the driving biogeochemical processes near the surface as compared to near the seafloor, we computed regressions using the mean of the two measurements. The regression results suggest warming has a large influence: the surface mean DO changes were more sensitive to the temperature change (−3.7 μmol kg^−1^°C^−1^) than to salinity (−0.98 μmol kg^−1^ PSU^−1^). In principle, assuming all other processes unchanged, the observed warming of surface mean temperatures at an average rate of ~0.020°C yr^−1^ over the 2000–2019 period corresponded to a reduction of the DO saturation concentration by 0.07~0.08 μmol kg^−1^ yr^−1^. Similarly, the slight salinity increase from 2005 to 2019 (0.0363 PSU yr^−1^) decreased DO by less than 0.06 μmol kg^−1^ yr^−1^. These predicted DO decreases from temperature and salinity effects on solubility were much smaller than the observed surface DO temporal trend: −0.69 μmol kg^−1^ yr^−1^, which can only be attributed to the decreasing primary productivity, affirmed by the impacts from decreasing surface chlorophyll. Overall, these results confirm our hypothesis (H1a) that the surface DO was mainly impacted by primary productivity change while solubility changes resulting from physical condition change are of secondary importance.

#### 4.1.2 Bottom DO is influenced by warming but mainly controlled by reduced respiration

It is intriguing to observe the opposing DO trends between surface and bottom. While the bottom water exhibits a faster warming rate of 0.041°C per year, it also demonstrates a significant increase in DO by 0.43 μmol kg^−1^ yr^−1^ per year. In theory, the faster warming in the bottom water should decrease the DO solubility at a rate of 0.15 ~0.20 μmol kg^−1^ yr^−1^ using the slopes between DO anomalies and temperature anomalies in Figure 7A. Thus, there must be a biological process, surpassing the anticipated decrease in DO due to warming at a rate of 0.15 to 0.20 μmol kg^−1^ yr^−1^. In other words, the reduction in labile organic carbon respiration had increased bottom DO by 0.6 μmol kg^−1^ yr^−1^ to explain the observed DO increase rate (0.43 μmol kg^−1^ yr^−1^), supporting hypothesis H1b. This 0.6 μmol kg^−1^ yr^−1^ rate of DO increase due to reduction of organic carbon respiration is almost the same as the observed DO decrease in surface water (−0.69 μmol kg^−1^ yr^−1^), due to the decrease of primary production (Section 4.1.1). Were this annual rate (0.6 μmol kg^−1^ yr^−1^) the only influence, the bottom DO could have increased about ~9 μmol kg^−1^ (about 0.2 mg L^−1^) over 15 years.

### 4.2 Processes impacting long-term carbon chemistry changes

#### 4.2.1 Surface pH is mainly controlled by variability of biological and freshwater processes

The regressions showed that pH was positively related to chlorophyll and salinity, but had no correlation with temperature. The positive correlation between surface pH and chlorophyll (Figure 7F) was consistent with the tight relation between biological carbon removal and pH in other coastal ecosystems, such as in the Salish Sea and two NE Pacific estuaries (Lowe et al., 2019). To better understand other processes, we next examine the sensitivity of pH to water mass mixing.

**FIGURE 8:**
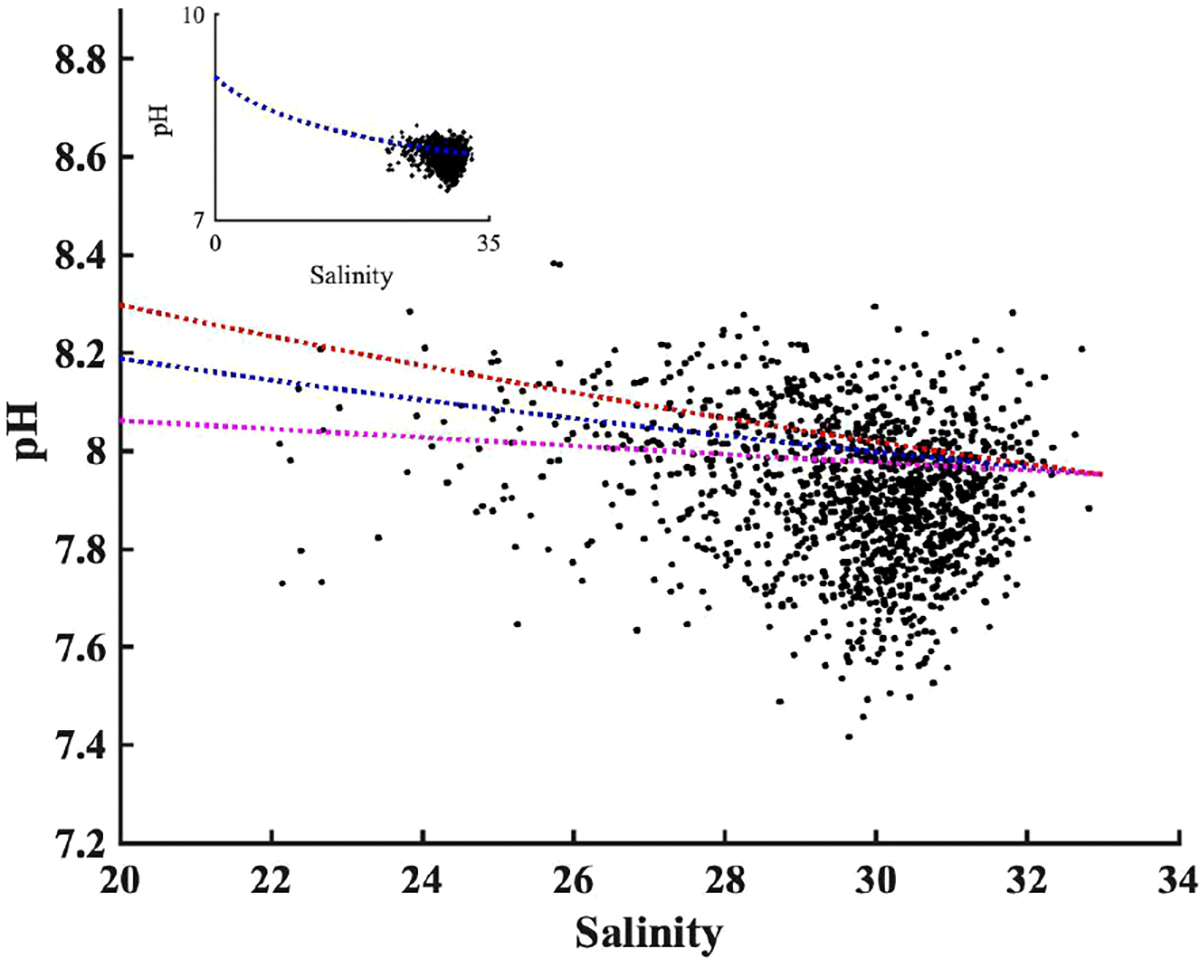
The observed pH values distribution along salinity after being adjusted to bay mean annual temperature (17°C). The blue dashed line shows the pH mixing curve @17°C, calculated using TA and DIC from measured salinity empirical regressions against salinity from Pimenta et al. (2023). The orange and purple dashed lines show the uncertainty in the mixing curve due to 10% changes in freshwater DIC endmember based on Pimenta et al. (2023)’s DIC vs salinity curve. The insert panel is the same pH versus salinity plot but with different axis limit, to make the zero-salinity endmember visible.

The TA: DIC ratio is the proxy of buffer capacity, i.e., the higher TA: DIC, the higher pH. We determined a “pH mixing curve” for pH as a function of salinity (blue line, Figure 8), to be used for two-endmember water mass mixing interpretations, as follows: we computed pH using CO2sys with TA and DIC inputs, from the salinity-TA (TA= 51.99 * salinity+477.62) and salinity-DIC (DIC=50.59*salinity+397.65) relationships of Pimenta et al. (2023), with mean temperature (17°C). Note that, unlike total alkalinity, DIC can be heavily impacted by biological activities; the pH mixing curve excludes influences of DIC production or consumption, and fluxes of CO_2_ across the air-sea interface. The wide range of the pH observations against salinity, deviations from the two end-members mixing model, confirm the importance of biological processes on pH observation. The overall agreement between observed pH and pH estimated by the two end-member mixing curve indicates the extent to which water mass mixing controls pH. The water mass mixing pH curve was sensitive to the freshwater endmember selection; a 10% DIC endmember change (± 40 μmol kg^−1^) has important impacts on absolute pH values (Figure 8), particularly in the low salinity range (S<25).

The freshwater endmember has TA: DIC of 1.2, higher than the TA: DIC of 1.06 for the S=33 salty endmember. This decreasing TA: DIC as salinity increases led to a decrease in pH with salinity, consistent with the observation in the New River Estuary (Van Dam and Wang, 2019). Because TA: DIC in Narragansett Bay was greater than 1, there was no minimum buffer capacity when TA: DIC is 1:1 zone as reported in other systems (Hu and Cai, 2013). In other words, the TA inputs from the watershed, in excess of DIC loads, related to the extreme precipitation events, helped buffer CO_2_-driven acidification in low-salinity regions.

For salinity<25 freshwater mixing can significantly impact surface pH, and for salinity>25 its impact is more modest. Therefore, the freshening between 2017 to 2019 (−3 PSU in Figure 6A), due to extreme precipitation events and increasing rivers discharge could increase pH by 0.05 using the slope between pH anomalies and salinity anomalies (0.0163 pH units per salinity unit) in Figure 7E. Though minor, it was still faster than the combined pH decreases caused by atmospheric CO_2_ dissolution (open ocean rate: −0.001~ −0.002 yr^−1^) and declines in primary productivity (−0.001~ −0.002 yr^−1^). The latter rate is determined by the DO (or Chl) decreasing rates in Figure 6 and the slopes between pH and DO (or Chl) in Figure 7.

While it may initially appear that the pH changes in Figure 8 contradict the positive relationship between pH anomaly and salinity anomaly in Figure 7E, it is crucial to consider the underlying spatial and temporal variability. Figure 8 summarized the impact of physical mixing and biological process on pH over much a wider salinity ranges (0 to 32): the saltier endmember typically experiences less nutrient loading from the watershed and lower primary productivity and respiration (resulting in a lower pH range), compared to the low salinity region characterized by high primary productivity and more intense respiration (or a higher pH range). In contrast, the positive correlation between pH anomaly and salinity anomaly in Figure 7E reflects diverse biological conditions within a narrow salinity anomaly (−2 to 2) across the entire bay. This relationship indicated that Narragansett Bay tended to be more heterotrophic (or lower pH) when it receives more freshwater. Consequently, extreme precipitation events in Narragansett Bay present a complex interplay: they increase the buffer capacity through water mass mixing, yet the resultant heterotrophic conditions, marked by enhanced respiration, lower the pH, highlighting the delicate balance between abiotic mixing and biological activities.

Interestingly, even though the pH anomalies were positively related to Chl and DO anomalies in Figures 7F, G, and both presented declining trends in the surface layer, there was no such decline in pH. In other words, the inter-annual variations could be correlated, but the long-term trends were not significant because the carbonate system was also sensitive to the buffer capacity change resulting from water mass mixing (Figure 7E). The observed surface pH change rejected Hypothesis H2a that surface OA was dominated by atmospheric CO_2_ dissolution and warming. In summary, the freshwater dynamics has heavily impacted the pH variability, which is consistent with previous studies that local physical condition can heavily modify regional OA changes (Salisbury and Jönsson, 2018; Wanninkhof et al., 2015). This natural variability can mask the atmospheric CO_2_ introduced OA and potential changes from primary productivity change, due to the sensitivity of buffer capacity to the water mass mixing. Future study with high precision freshwater end members and their variability will be helpful to quantify how future OA responds to freshwater dynamics, which likely will be further impacted by land use change and climate change.

#### 4.2.2 Bottom pH increased slightly primarily because of reduced organic carbon respiration

The bottom pH significantly increased, but the rate was minor (0.002 yr^−1^), supporting our hypothesis H2b. At least four processes were simultaneously affecting the changes in bottom water pH:

1. The water mass mixing model in section 4.2.1 also applied to the bottom conditions. The decreasing salinity between 2017 to 2019 (~1.5 PSU) can increase pH by about 0.025 during this three year time window. However, the overall slight salinity change can only increase pH by 0.0003 yr^−1^.
2. Decreasing organic respiration can relieve bottom OA by depressing the oxygen consumption. For example, as biological activities increased DO by a total ~9 μmol kg^−1^ (see section 4.1.2), the pH should increase about 0.014 (0.00156*9, in Figure 6G) over 15 years.
3. Even though the regression in Figure 6D shows there was no significant relationship between carbonate chemistry and temperature, partial pressure of CO_2_ increases about 4.3% with 1°C warming (Takahashi et al., 2002). This warming moved the net OA reaction to the right (CO_2_ +CO_3_^2−^+H_2_O->2HCO_3_^−^) or pH decreased about 0.015 per 1°C warming for a typical Narragansett Bay water (T=17°C, S=30, TA=2037 μmol kg^−1^, DIC=1915 μmol kg^−1^). So, in principle, the average 0.041°C yr^−1^ warming in the bottom can decrease pH by 0.0006 yr^−1^.

Overall, a weak but significant long-term pH increase was observed (Figure 6G). We attribute the increase to the reduced organic respiration at depth (the second process just described). However, this process alone would cause a faster pH increase than the rate was observed, so other three processes were important in limiting pH change in the bottom layer.

## 5 Summary and implications

Although the impacts of eutrophication and climate change on biogeochemical processes have been studied in various estuaries, to our knowledge there has not been targeted research focused on how they are affected when management goals of nutrient reduction are achieved. Narragansett Bay is one of a relatively small number of systems where aggressive reductions have been implemented, providing an opportunity to quantify ecosystem responses to the nutrient reductions within the context of climate change.

**FIGURE 9:**
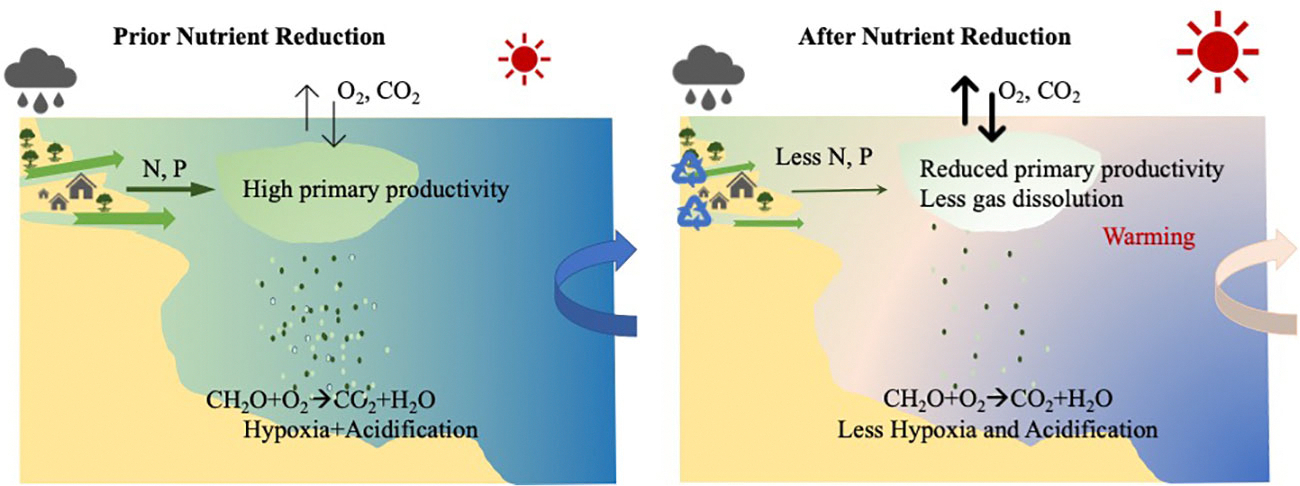
The conceptual diagram of the drivers of and interactions between nutrient reduction, hypoxia and ocean acidification in Narragansett Bay. Rapid local warming reduces oxygen solubility. The decreased amounts of anthropogenic nutrient inputs reduced the primary productivity and relieved the organic matter respiration (hypoxia and OA) in the bottom water. The hydrological changes from river discharge further modified the surface pH changes driven by air-sea CO_2_ exchange.

We examined the potential impacts of temperature, salinity and chlorophyll changes on annual mean DO and pH in both surface and bottom layers, focusing mainly on bay-wide means. We found that salinity had strong interannual variability, reaching its maximum between 2014 to 2017 and then decreasing. Bottom water is warming faster than surface water, both at rates higher than global mean warming. As bay-wide chlorophyll decreased due to nutrient reduction, the surface DO decreased and bottom DO increased (Figure 9). There was no significant pH change in the surface, reflecting complicated hydrological condition changes and the opposing effects of nutrient reduction and atmospheric CO_2_ dissolution. However, the bottom pH significantly increased probably because of reduced organic matter respiration at depth resulting from reduced primary productivity. This study provides a statistical examination of the water quality change in a “textbook” coastal region under intentional nutrient reduction and rapid climate change. Coastal areas undergoing similar reduction in nutrient loads are likely to experience beneficial increases in DO and pH. However, the impact of nutrient reduction on surface pH was minimal and may be masked by other biogeochemical processes; it may take years before any significant pH changes emerge from the background variabilities. Future high-quality data collection is still needed to further distinguish each process’s impact on water column pH change.

These results suggest that similar coastal estuaries may expect to see improvements in water quality as a result of reduced nutrient input such as in North Carolina New River (Mallin et al., 2005), Tampa Bay (Greening et al., 2014), Boston Harbor (Taylor et al., 2020), and Danish estuary (Staehr et al., 2017). Such enhancements are beneficial to the ecological balance, potentially fostering a more resilient aquatic environment in the face of ongoing climate shifts. However, it is important to note that the impact on pH may be overshadowed by the array of other biogeochemical processes at play. This subtlety indicates that observable pH changes may take considerable time to manifest against the backdrop of natural variability. Hence, estuaries elsewhere undertaking nutrient reduction should be cognizant of the time scales over which positive changes become detectable.

The case of Narragansett Bay serves as a precursor, implying that while immediate and clear-cut changes to water quality parameters such as pH may not always be apparent, the gradual and cumulative benefits of nutrient reduction are valuable. For other estuaries embarking on a similar path, continuous, high-resolution monitoring remains indispensable. Such data will not only reinforce our understanding of ecosystem responses to human interventions but also help isolate specific impacts of nutrient load adjustments on the water column’s water quality over extended periods. As global climate patterns evolve and anthropogenic impacts are mitigated, the knowledge gleaned will be vital in guiding future conservation and restoration efforts across diverse marine systems.

## Supplementary Material

Supplement1

## Data Availability

Publicly available datasets were analyzed in this study. This data can be found here: https://dem.ri.gov/environmental-protection-bureau/water-resources/research-monitoring/narraganset-bay-assessment-0.
