# Supplementary material for: Effect of nutrient reductions on dissolved oxygen and pH: a case study of Narragansett bay
Source: Front Mar Sci. Author manuscript; Available in PMC 2025 May 23. (PMC11960733; doi:10.3389/fmars.2024.1374873)
Supplement: Supplement1 [file NIHMS2002876-supplement-Supplement1.pdf]

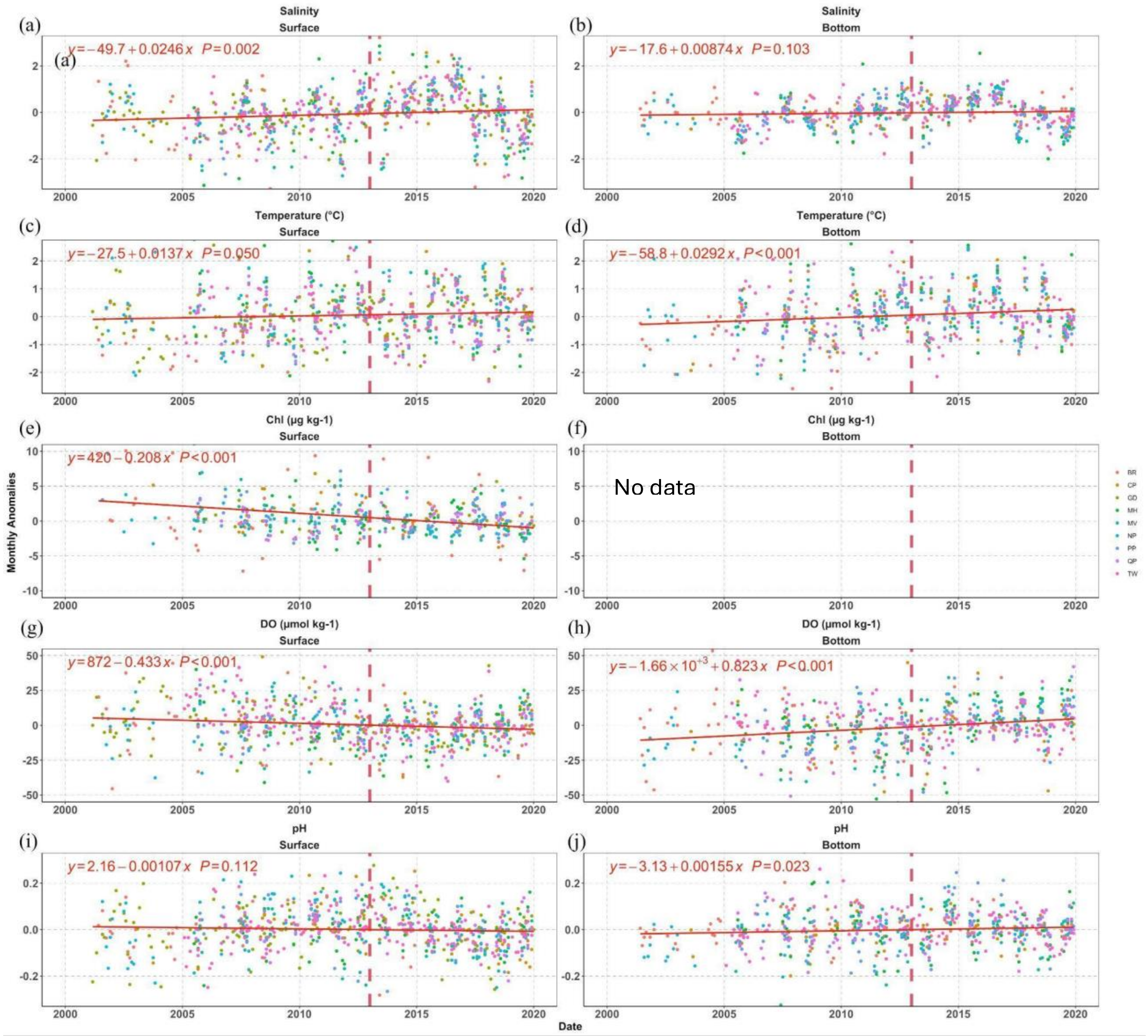

Figure S1 The time series of individual sites monthly anomalies for salinity (a, b), temperature (c,d), chlorophyll (e, f), DO (g, h), and pH (i, j). The left and right panels are for surface, and bottom, respectively. The red lines are the best fitted linear curves, and insert equations are the best fitted linear regression. The dash line in each panel shows the 2013 year when the 50% of nitrogen load reductions was completed.
